# Parkinson’s Disease Is Associated With Dysregulation of Circulatory Levels of lncRNAs

**DOI:** 10.3389/fimmu.2021.763323

**Published:** 2021-11-11

**Authors:** Kasra Honarmand Tamizkar, Pooneh Gorji, Mahdi Gholipour, Bashdar Mahmud Hussen, Mehrdokht Mazdeh, Solat Eslami, Mohammad Taheri, Soudeh Ghafouri-Fard

**Affiliations:** ^1^ Phytochemistry Research Center, Shahid Beheshti University of Medical Sciences, Tehran, Iran; ^2^ Men’s Health and Reproductive Health Research Center, Shahid Beheshti University of Medical Sciences, Tehran, Iran; ^3^ Department of Pharmacognosy, College of Pharmacy, Hawler Medical University, Erbil, Iraq; ^4^ Neurophysiology Research Center, Hamadan University of Medical Sciences, Hamadan, Iran; ^5^ Dietary Supplements and Probiotic Research Center, Alborz University of Medical Sciences, Karaj, Iran; ^6^ Department of Medical Biotechnology, School of Medicine, Alborz University of Medical Sciences, Karaj, Iran; ^7^ Skull Base Research Center, Loghman Hakim Hospital, Shahid Behehsti University of Medical Sciences, Tehran, Iran; ^8^ Institute of Human Genetics, Jena University Hospital, Jena, Germany; ^9^ Department of Medical Genetics, School of Medicine, Shahid Behehsti University of Medical Sciences, Tehran, Iran

**Keywords:** Parkinson’s disease, lncRNA, HULC, PVT1, MEG3, SPRY4-IT1, LINC-ROR, DSCAM-AS1

## Abstract

Long non-coding RNAs (lncRNAs) have been recently reported to be involved in the pathoetiology of Parkinson’s disease (PD). Circulatory levels of lncRNAs might be used as markers for PD. In the present work, we measured expression levels of *HULC*, *PVT1*, *MEG3*, *SPRY4-IT1*, *LINC-ROR* and *DSCAM-AS1* lncRNAs in the circulation of patients with PD *versus* healthy controls. Expression of *HULC* was lower in total patients compared with total controls (Expression ratio (ER)=0.19, adjusted P value<0.0001) as well as in female patients compared with female controls (ER=0.071, adjusted P value=0.0004). Expression of *PVT1* was lower in total patients compared with total controls (ER=0.55, adjusted P value=0.0124). Expression of *DSCAM-AS1* was higher in total patients compared with total controls (ER=5.67, P value=0.0029) and in male patients compared with male controls (ER=9.526, adjusted P value=0.0024). Expression of *SPRY4-IT* was higher in total patients compared with total controls (ER=2.64, adjusted P value<0.02) and in male patients compared with male controls (ER=3.43, P value<0.03). Expression of *LINC-ROR* was higher in total patients compared with total controls (ER=10.36, adjusted P value<0.0001) and in both male and female patients compared with sex-matched controls (ER=4.57, adjusted P value=0.03 and ER=23.47, adjusted P value=0.0019, respectively). Finally, expression of *MEG3* was higher in total patients compared with total controls (ER=13.94, adjusted P value<0.0001) and in both male and female patients compared with sex-matched controls (ER=8.60, adjusted P value<0.004 and ER=22.58, adjusted P value<0.0085, respectively). ROC curve analysis revealed that MEG3 and LINC-ROR have diagnostic power of 0.77 and 0.73, respectively. Other lncRNAs had AUC values less than 0.7. Expression of none of lncRNAs was correlated with age of patients, disease duration, disease stage, MMSE or UPDRS. The current study provides further evidence for dysregulation of lncRNAs in the circulation of PD patients.

## Introduction

As a progressive neurodegenerative condition, Parkinson’s disease (PD) affects 2-3% of the whole population age more than 65 years with a gradually increasing incidence (1). This disorder is characterized by resting tremor, bradykinesia, rigidity of muscles, balance disturbances, postural instability and a number of non-motor manifestations, particularly cognitive dysfunction which affects the vast majority of PD patients (2). PD is associated with alteration of expression and activity of several genes, particularly those related with dopamine-dependent oxidative stress (3). Many genetic and environmental risk factors of PD converge in pathways inducing cell death in dopaminergic neurons. In fact, high level of dopamine in cytoplasm of nigral neurons has been associated with dopamine oxidation and production of reactive oxygen species which have detrimental effects on these neurons (3). Cumulatively, dopamine-associated oxidative stress, dysfunction of synaptic vesicles and misfolding of α-synuclein produce an extending vicious cycle which perpetually results in death of dopaminergic neurons (3). PD has been associated with dysregulation of several transcripts among them are long non-coding RNAs (lncRNAs) (4). LncRNAs have possible role in brain development. A multi-disciplinary study of four highly conserved and brain-expressed lncRNA has shown that lncRNAs are functional transcripts with important roles in the development of vertebrate brain. This speculation is based on the observed preservation of lncRNAs across various amniotes, obvious conservation of their exons structures, and resemblances in lncRNA signature throughout the embryonic and early postnatal phases (5).

A number of lncRNAs affect pathoetiology of PD. For instance, *NEAT1* has been shown to promote the MPTP-associated autophagy in PD *via* increasing the stability of PINK1 protein (6). Moreover, *HOTAIR* has been found to target miR‐126‐5p to facilitate progression of PD *via* RAB3IP (7). A recent study has reported lower plasma levels of *MEG3* in PD patients compared with control group. Notably, authors have reported negative correlations between *MEG3* levels and Hoehn & Yahr (H&Y) stage and Non-Motor Symptoms Scale (NMSS) score in PD group. However, expression of this lncRNA has been positively correlated with Mini-Mental State Examination (MMSE) and Montreal Cognitive Assessment (MoCA) scores. Thus, authors have suggested close relation between *MEG3* expression and worsening of non-motor symptoms, cognitive impairments, and PD stage (8).

In the present work, we measured expression levels of *HULC*, *PVT1*, *MEG3*, *SPRY4-IT1*, *LINC-ROR* and *DSCAM-AS1* lncRNAs in the circulation of patients with PD *versus* healthy controls. These lncRNAs have been suggested to affect immune responses and participate in the pathoetiology of immune-related disorders of nervous system (9). Moreover, expressions of *LINC-ROR*, *MEG3* and *SPRY4-IT1* have been shown to be higher in patients with schizophrenia compared with healthy subjects (10). These lncRNAs might also affect pathoetiology of PD, since they can influence fundamental processes in this disorder such as autophagy. For instance, *HULC* has been found to target ATG7 (11), an autophagy related gene with crucial functions in the development of PD (12). Moreover, *PVT1* can induce cytoprotective autophagy (13). *MEG3* triggers autophagy through modulation of activity of ATG3 (14). The role of *LINC-ROR* in regulation of autophagy has been investigated in the context of cancer (15). These lncRNAs might also affect neurotoxic events. For instance, *SPRY4-IT1* has been shown to modulate ketamine-associated neurotoxicity in human embryonic stem cell-originated neurons (16). Besides, *DSCAM-AS1* has interaction with hnRNPL (17), an RNA-binding protein with possible role in the etiology of PD (18). However, their role of the development of PD has been less studied.

## Materials and Methods

### Patient and Controls

The present project was performed using the blood specimens collected from 50 cases of PD (Female/male ratio: 13/37) and 58 healthy individuals (Female/male ratio: 20/38). Patients were enlisted during January 2020-April 2021 from Farshchian, Hamadan, Iran. PD cases were diagnosed based on criteria proposed by the International Parkinson and Movement Disorder Society (19). Exclusion criteria were current or chronic infections, neoplastic conditions or any systemic disorder. H&Y staging system was used for evaluation of the functional disability associated with PD (20). Moreover, the MMSE was used as a screening tool for PD dementia, with values below 26 showing possible dementia (21). Moreover, Unified Parkinson’s Disease Rating Scale (UPDRS) was used as a rating tool to estimate the severity and progression of PD (22). Persons enlisted in the control group had no personal or family history of any neuropsychiatric disorder. The study protocol was confirmed by ethical committee of Shahid Beheshti University of Medical Sciences. All PD patients and controls signed the informed consent forms.

### Expression Assays

A total of 5 mL of peripheral blood was collected from PD patients and healthy persons in EDTA-blood collection tubes. Total RNA was extracted from these specimens using GeneAll extraction kit (Seoul, South Korea). The quality and quantity of RNA were assessed using gel electrophoresis and Nanodrop equipment. Afterwards, cDNA was made from roughly 75 ng of RNA using BioFact™ kit (Seoul, South Korea). The Ampliqon real time PCR master mix (Denmark) was used for making PCR reactions. Primers were designed so that the amplicon contains exon-intron boundary. Tests were accomplished in StepOnePlus™ RealTime PCR System (Applied Biosystems, Foster city, CA, USA). **Table 1** shows primers sequences. PCR program comprised a preliminary activation stage for 5 minutes at 94°C, and 40 cycles at 94°C for 15 seconds and 60°C for 45 seconds.

**Table 1:** Primer sequences.

Gene	Primer sequence		Primer length	Product size
HULC	Forward primer	ACGTGAGGATACAGCAAGGC	20	75
	Reverse primer	AGAGTTCCTGCATGGTCTGG	20	
PVT1	Forward primer	CCCATTACGATTTCATCTC	19	131
	Reverse primer	GTTCGTACTCATCTTATTCAA	21	
MEG3	Forward primer	TGGCATAGAGGAGGTGAT	18	111
	Reverse primer	GGAGTGCTGTTGGAGAATA	19	
SPRY4-IT1	Forward primer	AGCCACATAAATTCAGCAGA	20	115
	Reverse primer	GATGTAGGATTCCTTTCA	18	
LINC-ROR	Forward primer	TATAATGAGATACCACCTTA	20	170
	Reverse primer	AGGAACTGTCATACCGTTTC	20	
DSCAM-AS1	Forward primer	TCAGTGTCGCTACAGGGGAT	20	118
	Reverse primer	GGAGGAGGGACAGAGAAGGA	20	
B2M	Forward primer	AGATGAGTATGCCTGCCGTG	20	105
	Reverse primer	GCGGCATCTTCAAACCTCCA	20	

### Statistical Methods

The Statistical Package for the Social Sciences (SPSS) v.18.0 (SPSS Inc., Chicago, IL) was used for statistical assessments. Graphics were created using GraphPad Prism version 9.0 for Windows, GraphPad Software, La Jolla California USA. Expressions of lncRNAs in each sample were calculated using the Efficiency adjusted Ct of normalizer gene (B2M) - Efficiency adjusted Ct of target gene (comparative –delta Ct method). A two-way ANOVA was used to analyze effects of disease and gender on expression level of lncRNA in peripheral blood of patients and controls. Tukey *post hoc* test was used for multiple comparisons between subgroups. The “– delta Ct” Data in the figures were plotted as box and whisker plots (including the median [line], mean [cross], interquartile range [box], and minimum and maximum values. The delta delta Ct value was determined by subtracting the delta Ct of the control sample from the individual delta Ct of the test sample. The fold change of the test sample relative to the control sample was determined by 2^-delta delta Ct^ and was shown as lower limit-mean and upper limit in the figures and table. The correlations between transcript levels of lncRNAs were evaluated using regression model and Bonferroni correction for multiple comparisons. The partial correlation between expression levels and age of study participants, disease stage (Hoehn & Yahr stage), disease duration, MMSS and UPDRES was described by R and P values. The receiver operating characteristic (ROC) curves were depicted to appraise the diagnostic power of expression levels of lncRNAs. Youden’s J parameter was measured to find the optimum threshold. P value < 0.05 was considered as significant. The significance of difference in mean values of lncRNAs expression (mean of –delta Ct method) between two subgroups of patients using L-DOPA and other drugs was computed using the t-test. Dynamic principal component analysis of lncRNA expression profile was used to cluster samples *via* Gene Expression software (GenEx SW, Multid Analysis AB, Göteborg, Sweden). Normalized values were used for principal component analysis. Heatmaps were generated by using GenEx software.

## Results

### General Data of Cases

**Table 2** shows the clinical data and demographic information of PD cases.

**Table 2:** General data of cases.

Parameters	Groups		Values
Sex (number)	Male		37
	Female		13
Age [Years, mean ± SD (range)]	Male		69.64 ± 10.59 (47-89)
	Female		66.46 ± 12.6 (38-85)
Duration [Years, mean ± SD (range)]	Male		3.18 ± 3.65 (1-12)
	Female		5.38 ± 9.76 (1-36)
MMSE [mean ± SD (range)]	Male		22.84 ± 3.032 (17-29)
	Female		23.08 ± 2.499 (19-26)
UPDRS [mean ± SD (range)]	Male		23.92 ± 7.418 (13-41)
	Female		26.31 ± 9.437 (16-42)
Hoehn & Yahr stage (number)	I	Male	8
		Female	3
	II	Male	18
		Female	5
	III	Male	11
		Female	5
Drug administration (number)	L-DOPA		46
	Bromocriptine, Amantadine, Quetiapine		4

### Expression Assays

Expression of *HULC* was lower in total patients compared with total controls (Expression ratio (ER)=0.19, adjusted P value<0.0001) as well as in female patients compared with female controls (ER=0.071, adjusted P value=0.0004). Expression of *PVT1* was lower in total patients compared with total controls (ER=0.55, adjusted P value=0.0124). Expression of *DSCAM-AS1* was higher in total patients compared with total controls (ER=5.67, P value=0.0029) and in male patients compared with male controls (ER=9.526, adjusted P value=0.0024). Expression of *SPRY4-IT* was higher in total patients compared with total controls (ER=2.64, adjusted P value<0.02) and in male patients compared with male controls (ER=3.43, P value<0.03). Expression of *LINC-ROR* was higher in total patients compared with total controls (ER=10.36, adjusted P value<0.0001) and in both male and female patients compared with sex-matched controls (ER=4.57, adjusted P value=0.03 and ER=23.47, adjusted P value=0.0019, respectively). Finally, expression of *MEG3* was higher in total patients compared with total controls (ER=13.94, adjusted P value<0.0001) and in both male and female patients compared with sex-matched controls (ER=8.60, adjusted P value<0.004 and ER=22.58, adjusted P value<0.0085, respectively) (**Table 3**).

**Table 3:** The results of expression study of lncRNAs in peripheral blood of patients with PD compared with healthy controls.

lncRNAs		Total patients *vs.* Controls (50 *vs.* 58)	Male patients *vs.* Male Controls (37 *vs.* 38)	Female patients *vs.* Female Controls (13 *vs.* 20)	Female patients *vs.* Male patients 13 *vs.* 37)
*HULC*	Expression ratio (Lower Limit-Upper Limit)	0.19 (0.130-0.279)	0.513 (0.339-0.775)	0.071 (0.037-0.134)	0.962 (0.318-1)
	Adjusted P Value	<0.0001^*^	0.3746	0.0004	0.757
*PVT1*	Expression ratio (Lower Limit-Upper Limit)	0.55 (0.435-0.696)	0.619 (0.479-0.799)	0.49 (0.33-0.727)	0.962 (0.673-1.375)
	Adjusted P Value	0.0124^*^	0.2430	0.2770	0.999
*DSCAM-AS1*	Expression ratio (Lower Limit-Upper Limit)	5.672 (3.208-10.029)	9.526 (5.124-17.71)	3.375 (1.296-8.784)	0.116 (0.048-0.276)
	Adjusted P Value	0.0029^*^	0.0024^*^	0.5826	0.0683
*SPRY4-IT*	Expression ratio (Lower Limit-Upper Limit)	2.64 (1.735-4.019)	3.434 (2.174-5.432)	2.03 (1-4.106)	0.913 (0.482-1.728)
	Adjusted P Value	<0.0227	<0.0397^*^	<0.7471	0.9999
*LINC-ROR*	Expression ratio (Lower Limit-Upper Limit)	10.36 (6.236-17.21)	4.575 (2.634-7.948)	23.47 (10.014-55.024)	0.854 (0.395-1.848)
	Adjusted P Value	<0.0001^*^	0.0345^*^	0.0019^*^	0.9970
*MEG3*	Expression ratio (Lower Limit-Upper Limit)	13.94 (7.86-24.706)	8.603 (4.615-16.037)	22.58 (8.639-59.014)	1.392 (0.583-3.321)
	P-value	<0.0001	<0.0044^*^	<0.0085^*^	0.9811

The expression ratio of each gene (mean, lower limit and upper limit) is shown as the ratio of expression of the first group compared to the second group in each column. * shows significance.

**Figure 1:**
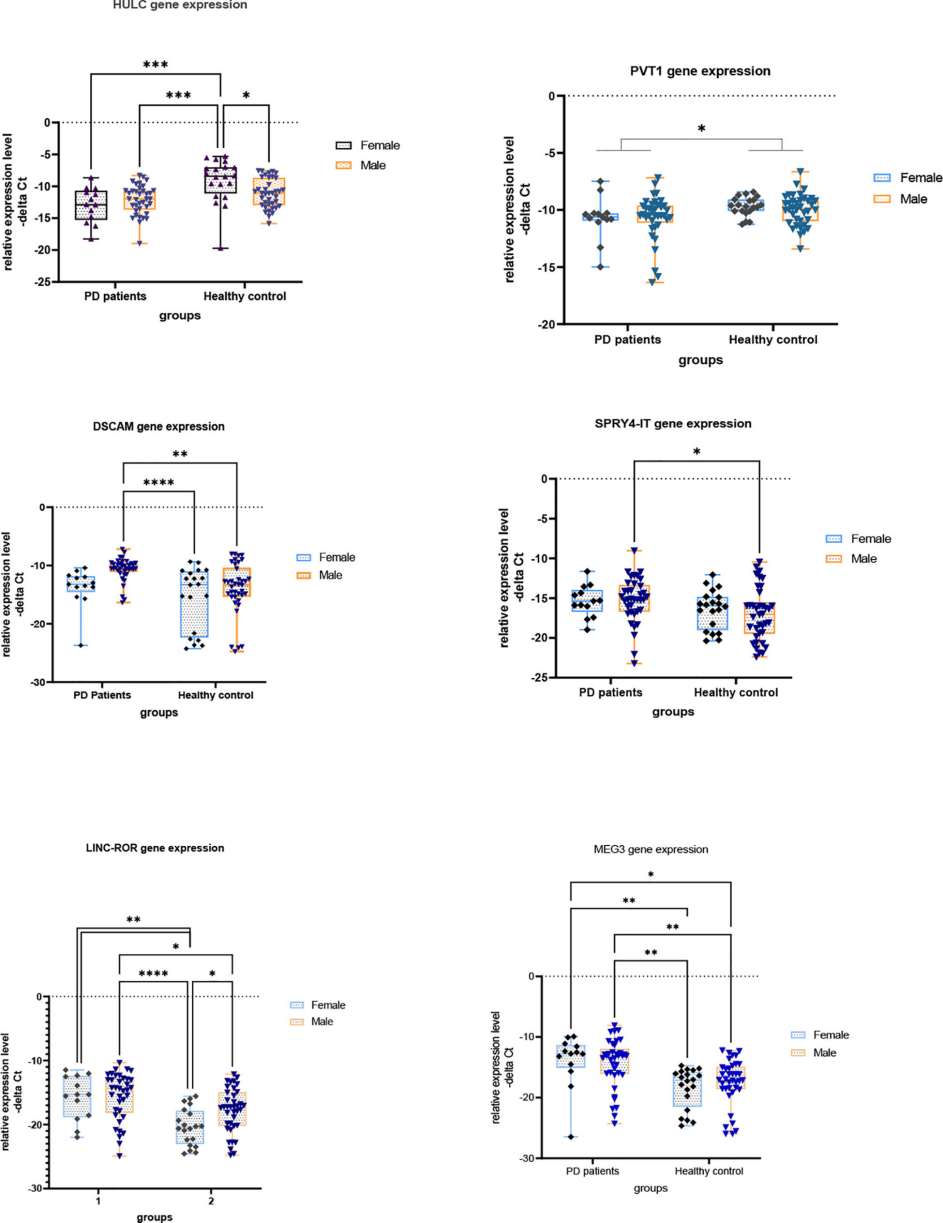
Relative expression levels of lncRNAs in PD patients *versus* controls (*P value < 0.05, **P value < 0.001, ***P < 0.001 and ****P value < 0.0001).

**Figure 2:**
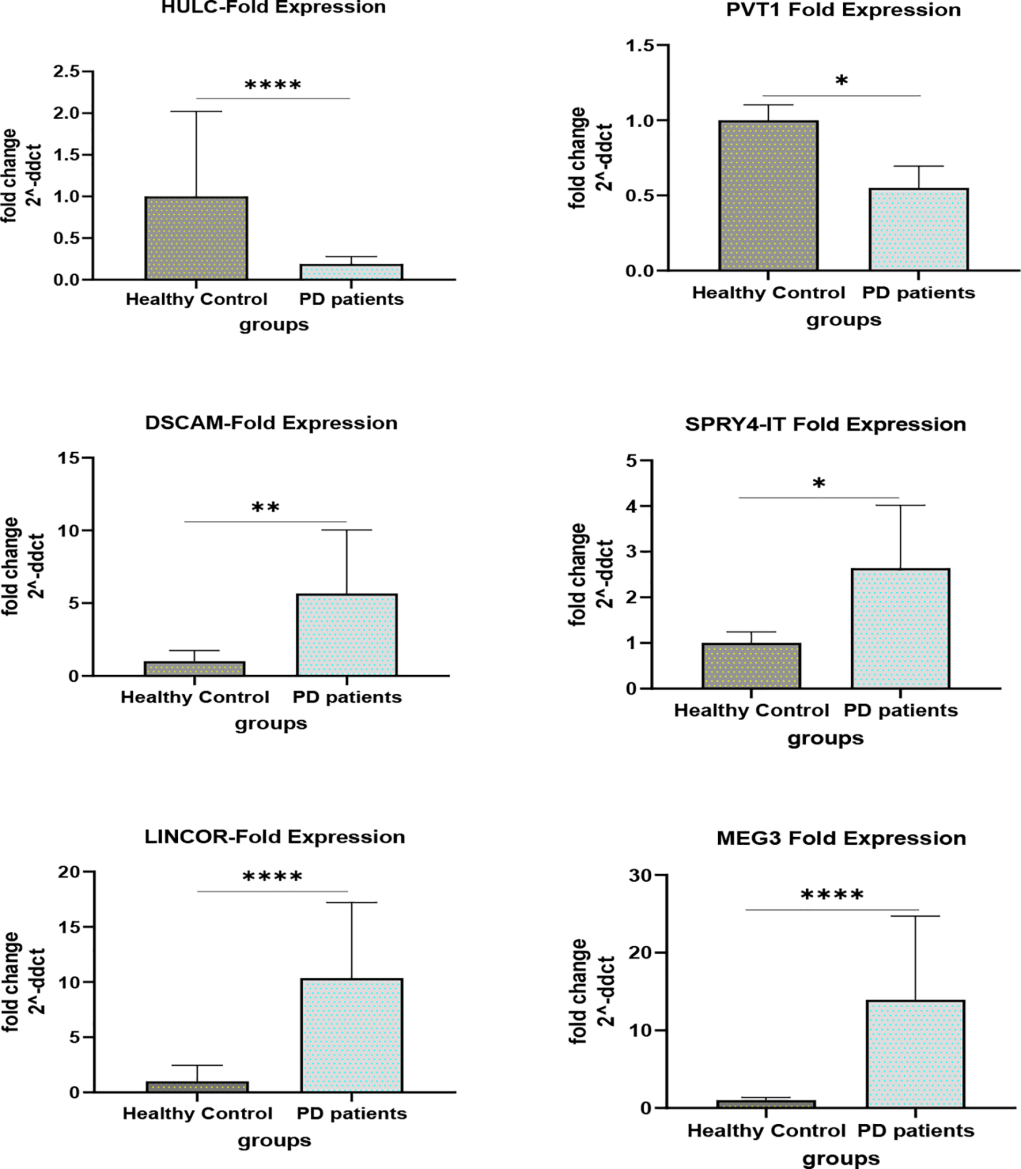
Fold changes of lncRNAs in PD patients *versus* controls (*P value < 0.05, **P value < 0.001 and ****P value < 0.0001).

**Figures 1** and **2** show relative expression of expression levels of lncRNAs and their fold changes in PD patients *versus* controls.

**Figure 3:**
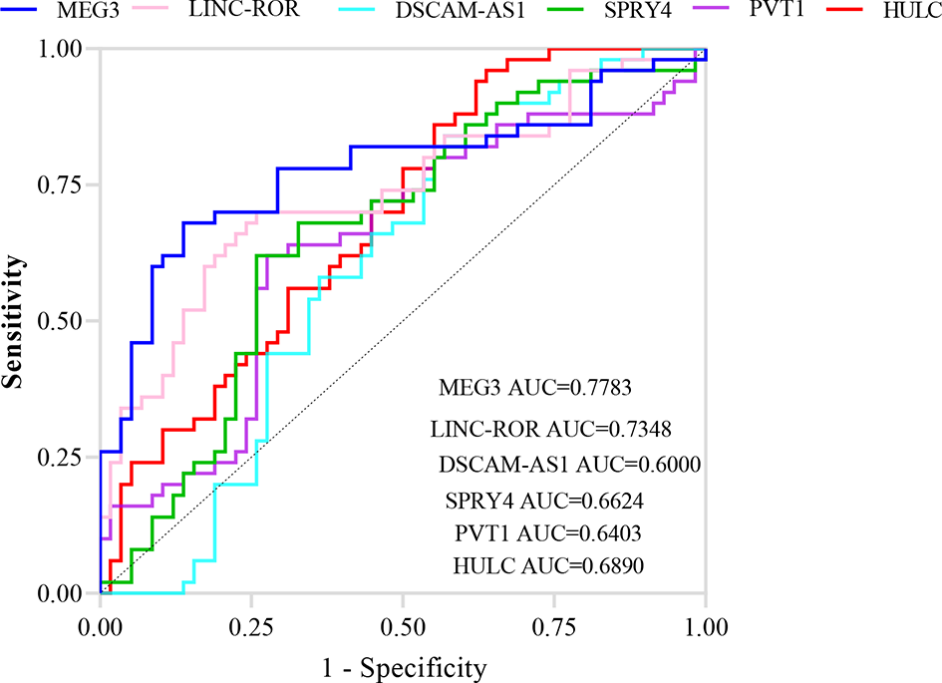
ROC curves showing the power of lncRNAs in separation of PD patients from controls.

ROC curve analysis revealed that MEG3 and LINC-ROR have diagnostic power of 0.77 and 0.73, respectively (**Figure 3**). Other lncRNAs had AUC values less than 0.7.

**Table 4** shows sensitivity, specificity and AUC values of each lncRNA in separation of PD cases from controls. This type of analysis was repeated for distinct sex-based groups. *HULC* and *PVT1* could differentiate only between female subgroups. On the other hand, *DSCAM-AS1* and *SPRY4-IT* could differentiate only between male subgroups.

**Table 4:** Sensitivity, specificity and AUC values of each lncRNA in separation of PD cases from controls.

	HULC				PVT1				DSCAM-AS1				SPRY4-IT				LINC-ROR				MEG3				
	AUC ± SD	Sensitivity	Specificity	P Value	AUC ± SD	Sensitivity	Specificity	P Value	AUC ± SD	Sensitivity	Specificity	P Value	AUC ± SD	Sensitivity	Specificity	P Value	AUC ± SD	Sensitivity	Specificity	P Value	AUC ± SD	Sensitivity	Specificity	P Value
Total patients *vs.* total normal controls (50 *vs.* 58)	0.68± 0.050	0.96	0.36	0.0007	0.64 ± 0.054	0.62	0.72	0.0122	0.6 ± 0.055	0.84	0.43	0.074	0.66 ± 0.052	0.62	0.74	0.0037	0.73 ± 0.049	0.7	0.74	<0.0001	0.77 ± 0.047	0.68	0.86	<0.0001
Female patients *vs.* Female normal controls (13 *vs.* 20)	0.84 ± 0.068	0.92	0.75	0.0009	0.74 ± 0.10	0.84	0.85	0.018	0.53 ± 0.10	0.92	0.3	0.76	0.62 ± 0.09	0.76	0.5	0.23	0.68 ± 0.06	0.70	0.63	0.005	0.85 ± 0.08	0.77	0.83	0.0006
Male patients *vs.* Male normal controls (37 *vs.* 38)	0.6 ± 0.006	0.22	0.94	0.13	0.59 ± 0.06	0.54	0.68	0.14	0.73 ± 0.06	0.86	0.73	0.0005	0.67 ± 0.06	0.83	0.5	0.0089	0.68 ± 0.062	0.70	0.63	0.0057	0.73 ± 0.059	0.75	0.68	0.0004

Expression of none of lncRNAs was correlated with age of patients, disease duration, disease stage, MMSE or UPDRS (**Table 5**).

**Table 5:** The results of partial correlation between expression of lncRNAs and age, Disease duration, Disease stage, MMSE and UPDRS [Controlled for sex, Diseases duration was classified into 3 ranges (1-5, 6-10 and more than 10 years)].

Parameters	Age		HULC		PVT1		DSCAM-AS1		SPRY4-IT		LINC-ROR		MEG3		Disease stage (Hoehn & Yahr stage)		Disease duration		MMSE		UPDRS	
	R	P value	R	P value	R	P value	R	P value	R	P value	R	P value	R	P value	R	P value	R	P value	R	P value	R	P value
Age	1	0	0.06	0.64	0.051	0.72	-0.079	0.58	-0.05	0.71	0.23	0.1	0.15	0.30	0.14	0.31	-0.09	0.49	-0.61*	0.000002	0.11	0.43
Disease duration	-0.09	0.49	-0.13	0.354	-0.07	0.63	-0.046	0.75	0.048	0.7385	-0.04	0.768	-0.01	0.91	0.6*	0.000004	1	0	-0.39*	0.005577	0.52*	0.0001
Disease stage (Hoehn & Yahr stage)	0.14	0.31	-0.05	0.7	0.007	0.96	-0.05	0.7	0.02	0.87	0.04	0.73	0.07	0.60	1	0	0.6*	0.000004	-0.54*	0.000048	0.70*	1.4167E-8
MMSE	-0.61	0.000002	-0.09	0.53	0.033	0.81	0.044	0.76	0.014	0.92	-0.13	0.37	-0.06	0.64	-0.5	0.000048	-0.39*	0.005577	1	0	-0.33*	0.02
UPDRS	0.11	0.43	-0.11	0.41	0.052	0.72	-0.005	0.970	-0.039	0.78	0.078	0.59	0.15	0.27	0.70*	1.4167E-8	0.52*	0.000103	-0.33*	0.02035	1	0

* shows significance.

Expressions of lncRNAs were significantly correlated with each other in both PD patients and controls (**Table 6**).

**Table 6:** Correlations between expressions of lncRNAs in study groups.

DSCAM-AS1	Controls	0.48*	0.0001									
	Patients	0.66*	<0.0001								
SPRY4-IT	Controls	0.45*	0.0004	0.63*	<0.0001						
	Patients	0.51*	0.0001	0.49*	0.0003						
LINC-ROR	Controls	0.31	0.0167	0.63*	<0.0001	0.57*	<0.0001				
	Patients	0.53*	<0.0001	0.45	0.001	0.55*	<0.0001				
MEG3	Controls	0.33*	0.0096	0.60*	<0.0001	0.42*	0.0008	0.61*	<0.0001		
	Patients	0.46*	0.0006	0.35	0.0123	0.25	0.0738	0.49*	0.0003		
PVT1	Controls	0.46*	0.0003	0.43*	0.0006	0.36	0.0051	0.47*	0.0002	0.44*	0.0006
	Patients	0.34	0.0138	0.40	0.0035	0.32	0.0207	0.58*	<0.0001	0.55*	<0.0001
		R	P Value	R	P Value	R	P Value	R	P Value	R	P Value
		HULC		DSCAM-AS1		SPRY4-IT		LINC-ROR		MEG3	

Correlations between expressions of lncRNAs in study groups (R values are presented; after correction for multiple comparisons (Bonferroni correction), P value less than 0.0016 was accepted as significant. * shows significance.

**Figure 4:**
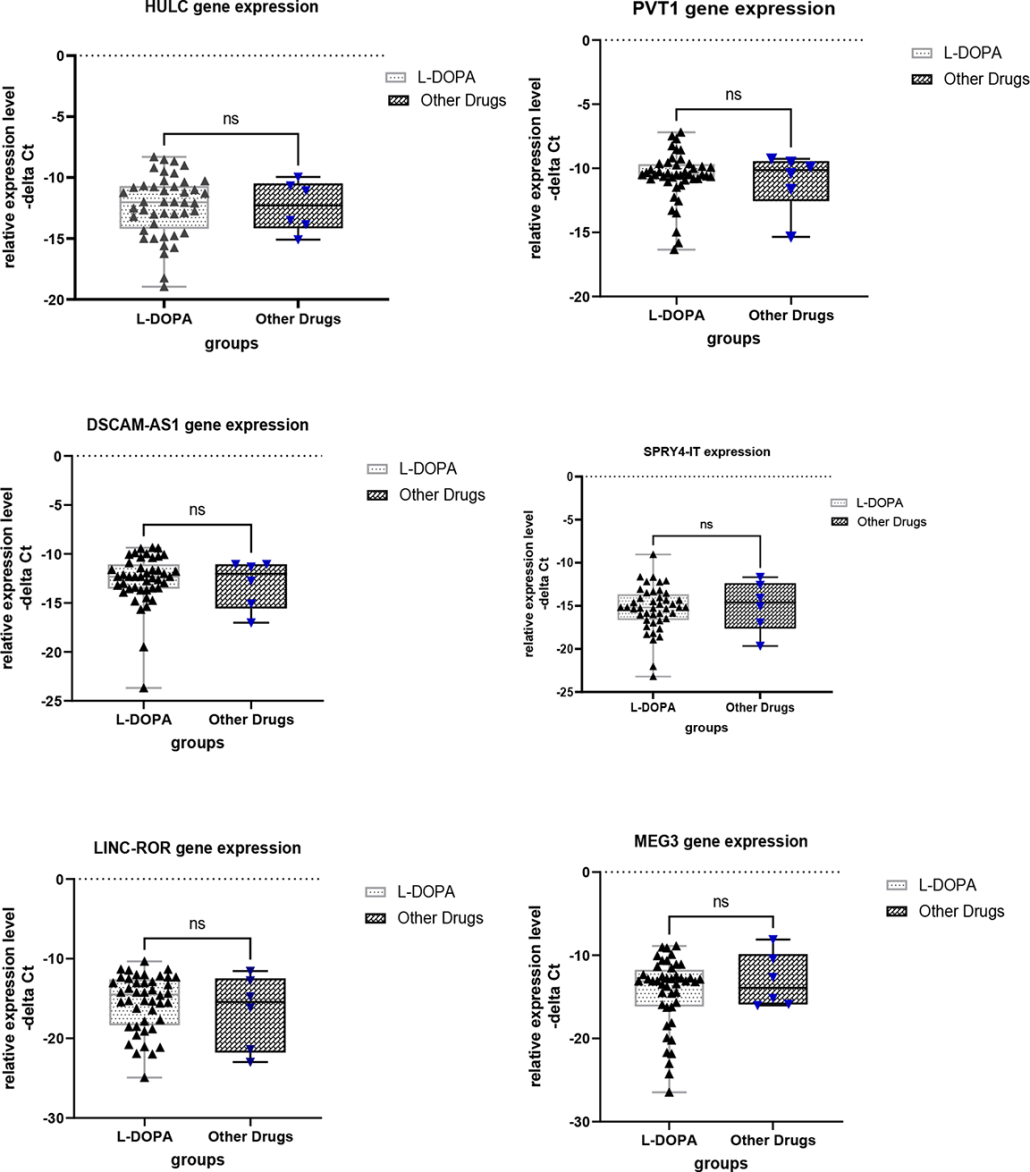
Comparison of expression levels of lncRNAs between patients receiving L-DOPA and those under treatment with other drugs. ns, not significant.

Finally, we compared expression levels of lncRNAs in patients receiving L-DOPA *versus* those being under treatment with other drugs (**Figure 4**). This analysis revealed no significant difference in expression of lncRNAs between these two groups.

**Figure 5:**
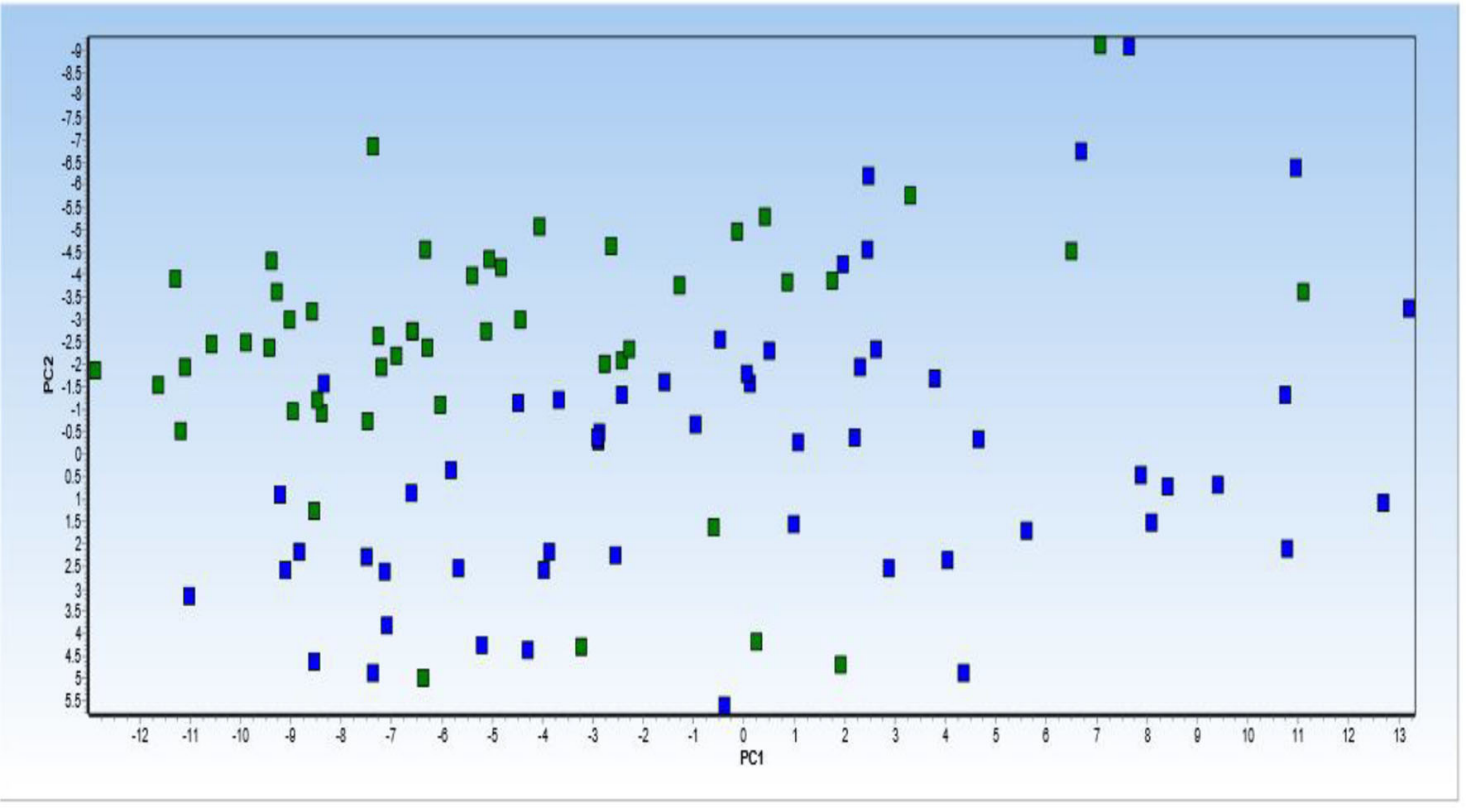
Principal component analysis (PCA) of 6 lncRNA expression profiles in patients with Parkinson diseases compared with healthy control. PCA of the 6 lncRNAs expression data could not clearly clusters samples collected from healthy controls (blue squares) and patients with Parkinson (green squares) into their respective groups. Normalized values were used for principal component analysis.

Principal component analysis (PCA) was performed on 6 lncRNA expression profiles in patients with PD compared with healthy control. PCA of the 6 lncRNAs expression data could not clearly clusters samples collected from healthy controls (blue squares) and patients with Parkinson (green squares) into their respective groups (**Figure 5**).

**Figure 6:**
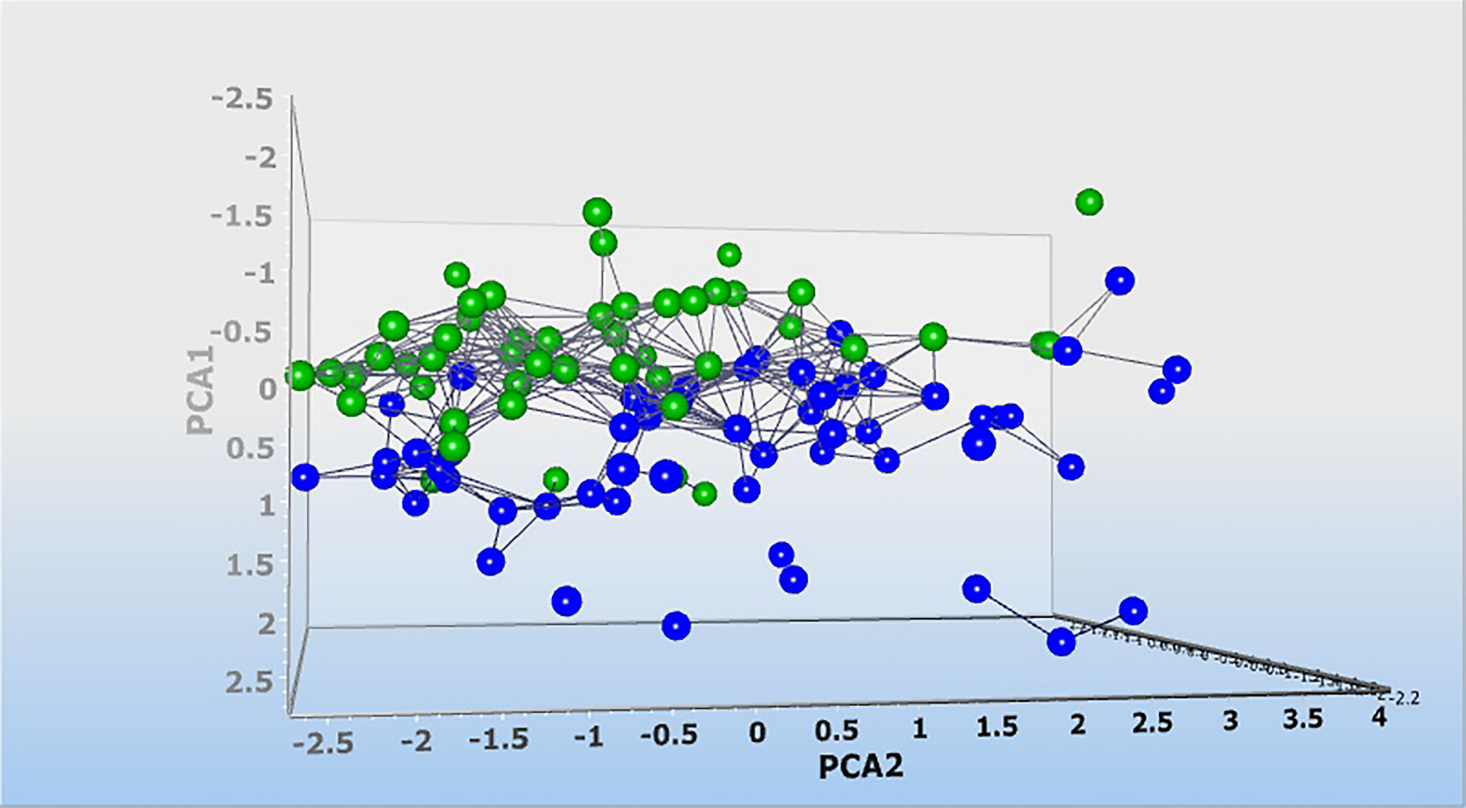
Dynamic principal component analysis (DPCA) of 6 lncRNA expression profiles. DPCA was used to filter out and exclude lncRNA with low standard deviation. LncRNA PVT1 was excluded and 5 lncRNA expression data were used to clusters samples collected from healthy controls (blue squares) and patients with Parkinson (green squares) into their respective groups. Normalized values were used for principal component analysis.

Then, dynamic principal component analysis (DPCA) was performed on the lncRNA results from the analyzed samples to determine how the 6 differentially expressed lncRNAs were distributed among the samples from PD patients and healthy controls. DPCA excluded lncRNA PVT1 with low standard deviation. Thus, 5 lncRNAs expression data were used to clusters samples collected from healthy controls (blue squares) and patients with PD (green squares) into their respective groups. As shown in **Figure 6**, the DPCA almost clearly separated the samples collected from healthy controls (blue squares) and patients with PD (green squares) into their respective groups.

**Figure 7:**
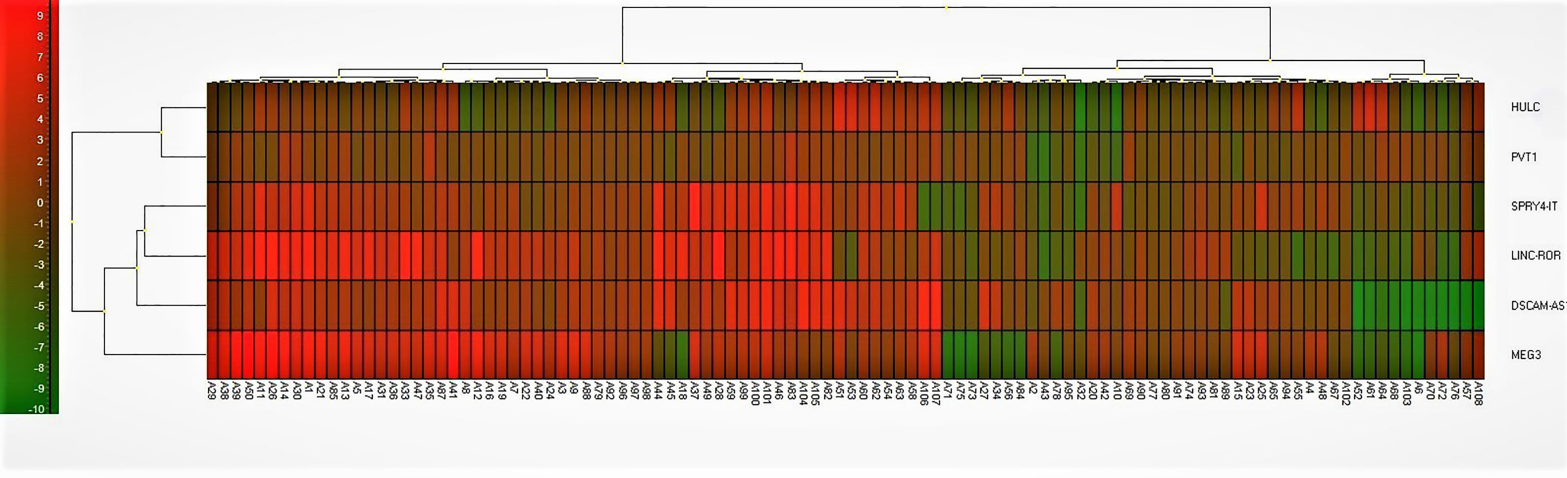
Log2 Fold Change Heat Map. A heat map for the subjects with Parkinson diseases and healthy control. Log2 fold change was calculated based on delta Ct value compared to the control samples. Red color implies increased expression while green implies decreased expression. LncRNAs on the right are clustered using a hierarchical clustering method (Ward’s method, Euclidean distances) and 5 clusters were found. Cluster 1 = HULC and PVT1; Cluster 2 = SPRY4-IT; Cluster 3 = LINC-ROR; Cluster 4 = DSCAM-AS1; Cluster 5 = MEG3. Most of patient samples (A1-A50) were located on the left side with increased expression of lncRNAs studied in this work.

Finally, we depicted Log2 Fold Change Heat Map for lncRNA levels (**Figure 7**). Most of patient samples (A1-A50) were located on the left side with increased expression of lncRNAs studied in this work.

## Discussion

In the present work, we measured expression levels of 6 lncRNAs in the circulation of patients with PD *versus* healthy controls. Expression of *HULC* was lower in total patients compared with total controls as well as in female patients compared with female controls. This lncRNA has a role in regulation of immune response, since up-regulation of *HULC* has been shown to has a necessary role in pro-inflammatory responses in the course of LPS-associated sepsis (23). In addition, *HULC* has a role in regulation of apoptosis. Experiments in the contexts of various neoplasms have indicated an anti-apoptotic role for *HULC* (24, 25). This function of *HULC* has not been assessed in neurons. If this lncRNA exerts similar role in neurons, down-regulation of *HULC* in the circulation of patients with PD might be associated with higher apoptosis of neurons. It has been widely accepted that apoptosis of nigral dopaminergic neurons has essential roles in the development of PD (26). Various mechanisms including both intrinsic and extrinsic routes participate in the degeneration of dopaminergic neurons in this disorder (26). However, the exact position of *HULC* within this complicated network of apoptosis-related mechanisms needs to be clarified.

Expression of *PVT1* was lower in total patients compared with total controls. *PVT1* silencing has been shown to induce apoptosis and inhibit cell cycle transition *via* modulating EFGR pathway (27). Experiment in animal model of PD has shown the impact of EGFR signaling in cell death of dopaminergic neurons in the course of neuro-apoptosis (28).

Expressions of *DSCAM-AS1* and *SPRY4-IT* were higher in total patients compared with total controls and in male patients compared with male controls. *DSCAM-AS1* has been previously reported as an Estrogen receptor α-dependent lncRNA with critical roles in the regulation of cell growth and migration (29). Since estrogen and some selective estrogen receptor modulators have been suggested as possible therapeutic options for PD (30), identification of the molecular mechanism of participation of *DSCAM-AS1* in the pathetiology of PD has clinical significance. The observed sex-biased dysregulation of this lncRNA among PD patients further support the interaction between estrogen receptor and this lncRNA. *SPRY4-IT1* has been shown to modulate ketamine-associated neurotoxicity in human embryonic stem cell-originated neurons *via* EZH2 (16). Up-regulation of this lncRNA in the circulatory blood of PD patients might be a compensatory mechanism to decrease PD-associated neuron loss.

Expressions of *LINC-ROR* and *MEG3* were higher in total patients compared with total controls and in both male and female patients compared with sex-matched controls. *LINC-ROR* has been shown to regulate apoptosis through influencing p53 ubiquitination *via* regulation of miR-204-5p/MDM2 axis (31). *MEG3* has been shown to affect neuron apoptosis through miR-181b-12/15-LOX signaling (32). Thus, modulation of apoptotic pathways is possible mechanism of participation of these lncRNAs in PD.

ROC curve analysis revealed that *MEG3* and *LINC-ROR* have diagnostic power of 0.77 and 0.73, respectively. Other lncRNAs had AUC values less than 0.7. Thus, *MEG3* and *LINC-ROR* are possible markers for PD.

Expression of none of lncRNAs was correlated with age of patients, disease duration, disease stage, MMSE or UPDRS. The current study provides further evidence for dysregulation of lncRNAs in the circulation of PD patients. Therefore, expression level of these lncRNAs is independent from PD course.

Moreover, the DPCA almost clearly separated the samples collected from healthy controls and patients with PD into their respective groups. This suggests that the observed lncRNA differences are associated with the pathophysiology of PD, and these lncRNA might constitute an important biomarker signature for PD.

In conclusion, the current study shows dysregulation of lncRNAs in the circulation of PD patients. The study has limitations regarding small sample size and lack of inclusion of drug-naïve patients. Moreover, it is important to characterize each lncRNA in detail, such as the structure and function of each lncRNA, and to quantify the role of lncRNA in PD in multinational multicenter studies.

## Data Availability Statement

The raw data supporting the conclusions of this article will be made available by the authors, without undue reservation.

## Ethics Statement

The study protocol was confirmed by ethical committee of Shahid Beheshti University of Medical Sciences. The patients/participants provided their written informed consent to participate in this study. Written informed consent was obtained from the individual(s) for the publication of any potentially identifiable images or data included in this article.

## Author Contributions

SG-F wrote the draft and revised it. MT and BH designed and supervised the study. SE analyzed the data. KH, MG, and PG performed the experiment. All authors contributed to the article and approved the submitted version.

## Funding

The current study was supported by a grant from Shahid Beheshti University of Medical Sciences.

## Conflict of Interest

The authors declare that the research was conducted in the absence of any commercial or financial relationships that could be construed as a potential conflict of interest.

## Publisher’s Note

All claims expressed in this article are solely those of the authors and do not necessarily represent those of their affiliated organizations, or those of the publisher, the editors and the reviewers. Any product that may be evaluated in this article, or claim that may be made by its manufacturer, is not guaranteed or endorsed by the publisher.
